# Gametocyte carriage in *Plasmodium falciparum*-infected travellers

**DOI:** 10.1186/1475-2875-12-31

**Published:** 2013-01-24

**Authors:** Catherine H Roberts, Margaret Armstrong, Ewa Zatyka, Samuel Boadi, Simon Warren, Peter L Chiodini, Colin J Sutherland, Tom Doherty

**Affiliations:** 1 Hospital for Tropical Diseases, Mortimer Market Centre, Capper Street, London, WC1E 6JB, UK; 2 Department of Clinical Parasitology, Hospital for Tropical Diseases, Mortimer Market Centre, Capper Street, London, WC1E 6JB, UK; 3 Faculty of Infectious & Tropical Diseases, London School of Hygiene and Tropical Medicine, Keppel Street, London, WC1E 7HT, UK

**Keywords:** Falciparum malaria, Gametocytes, Adult travellers

## Abstract

**Background:**

Gametocytes are the sexual stage of *Plasmodium* parasites. The determinants of gametocyte carriage have been studied extensively in endemic areas, but have rarely been explored in travellers with malaria. The incidence of gametocytaemia, and factors associated with gametocyte emergence in adult travellers with *Plasmodium falciparum* malaria was investigated at the Hospital for Tropical Diseases in London.

**Methods:**

Clinical, parasitological and demographic data for all patients presenting with *P. falciparum* malaria between January 2001 and December 2011 were extracted from a prospective database. These data were supplemented by manual searches of laboratory records and patient case notes.

**Results:**

Seven hundred and seventy three adult patients with laboratory-confirmed *P. falciparum* malaria were identified. Four hundred and sixty five (60%) were born in a country where malaria is endemic. Patients presented to hospital a median of four days into their illness. The median maximum parasite count was 0.4%. One hundred and ninety six patients (25%) had gametocytes; 94 (12%) on admission, and 102 (13%) developing during treatment. Gametocytaemia on admission was associated with anaemia and a lower maximum parasitaemia. Patients with gametocytes at presentation were less likely to have thrombocytopenia or severe malaria. Patients who developed gametocytes during treatment were more likely to have had parasitaemia of long duration, a high maximum parasitaemia and to have had severe malaria. There was no apparent association between the appearance of gametocytes and treatment regimen.

**Conclusions:**

The development of gametocytaemia in travellers with *P. falciparum* is associated with factors similar to those reported among populations in endemic areas. These data suggest that acquired immunity to malaria is not the only determinant of patterns of gametocyte carriage among patients with the disease.

## Background

Gametocytes are the sexual stage of malaria parasites. The generation of male and female gametes is necessary for transmission to the mosquito vector where sexual recombination and generation of new haploid progeny takes place. In acute infection with *Plasmodium falciparum*, gametocytes arise seven to 15 days after the initial patent parasitaemia and last for a mean of 6.4 (range 2.5–22) days in the circulation, longer than the typical duration of asexual parasitaemia [1]. Factors associated with gametocyte production include the local intensity of malaria transmission [2,3], the duration of infection, presence of anaemia [4] and other haematological factors such as reticulocyte and lymphocyte counts [5]. Gametocytes are of increasing academic interest as transmission-blocking interventions have been recognized as potentially important tools for malaria control and elimination [6-9].

Few data have been published on gametocyte carriage among travellers with *P. falciparum*. Travellers represent an interesting cohort for two reasons. First, they usually present early in the natural history of their disease, and second, they may have little immunity to malaria. This provides an opportunity to analyse the effect of other variables, such as the duration of infection and the impact of disease severity, on the likelihood with which gametocytes are seen. In this study, factors associated with gametocytaemia were assessed among a population of adult travellers with *P. falciparum* malaria at the Hospital for Tropical Diseases, London, UK.

## Methods

The Hospital for Tropical Diseases is a tertiary referral hospital in London. Patients may present directly, by general practitioner referral or as a transfer from another hospital. Demographic details of all patients admitted to the hospital with falciparum malaria are prospectively collected into a database. Patients treated as outpatients were identified by reviewing laboratory records. Further data were collected from laboratory and case note records. All patients diagnosed with *P. falciparum* between the beginning of January 2001 and end of December 2011 were included. The diagnosis of malaria was made by the UK’s Parasitology Reference Laboratory using standard thick and thin films which were, for those admitted, repeated daily until asexual parasites were no longer seen. Severe malaria was defined according to the unit’s guidelines: parasitaemia >2% and/or the presence of complications [10]. Complications were defined as: cerebral involvement, severe anaemia (Hb <8.0 g/dl), renal failure (creatinine >265 mmol/L), pulmonary oedema, hypoglycaemia, bleeding, disseminated intravascular coagulation and acidosis. The cut-off for severe malaria was a parasitaemia estimate of greater than 2%, by microscopy. This conservative definition, the established guideline for the study facility, is routinely deployed rather than the WHO criteria of 4% parasitaemia. Associations between pairs of binary variables were tested for significance using the c^2^ distribution. Continuous variables were compared across binary categories using the Wilcoxon rank-sum test. Multivariate logistic regression was performed on those (binary) variables found significantly associated with gametocyte carriage in the univariate tests. All statistical analyses were performed in STATA (version 11; Timberlake Consultants Limited, London, UK). The study was reviewed and approved by the Audit and Research Committee at HTD who stated that, as this was a retrospective case note review, further formal ethical approval was not required.

## Results

Eight hundred and twenty-two patients with laboratory-confirmed *P. falciparum* malaria were identified. Forty-nine of these were under the age of 18 years and were excluded as their biological response to malaria differs from adults. This left a total adult population of 773.

### Demographics

**Figure 1:**
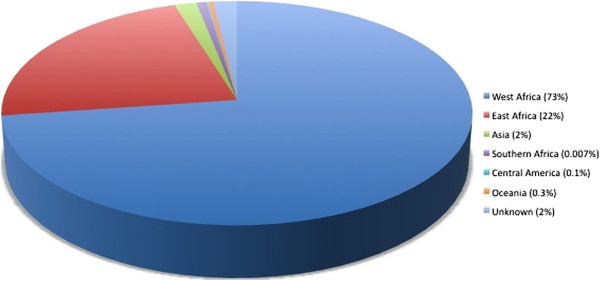
Area of travel.

**Figure 2:**
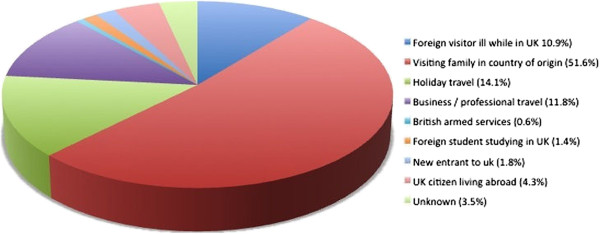
Reason for travel.

Median age was 38 years and 63% were male. Four hundred and sixty five (60%) had been born in a malaria-endemic country and may have had partial immunity to the disease. Two hundred and sixty nine (34.8%) had been born in a country without malaria transmission and were assumed to be malaria-naïve (Table 1). The remainder did not have a country of origin recorded. Most (741, 96%) had travelled to Africa (Figure 1) and had visited relatives in their country of origin (399, 52%) (Figure 2).

**Table 1:** Population characteristics

**Characteristics**	**N = 773 (%)**
Age, years, median (IQR)	38 (30–48)
Male	493 (63%)
UK born	208 (27%)
Admitted to hospital	758 (98%)
Time to presentation, days. median (IQR)	4 (2–7)
Median maximum parasite count (%), (IQR)	0.4% (0.05–2.0)
Severe malaria	364 (47%)

### Malaria infection

Relatively few patients had taken appropriate anti-malarial prophylaxis (Table 2). Median time from onset of symptoms to presentation was four days (IQR 2–7). The median maximum parasite count was 0.4% (IQR 0.05–2.00). Most (758, 98%) were admitted to hospital. A large proportion (364, 47%) had at least one marker of severe disease. One hundred and ninety six patients (25%) had gametocytes identified in peripheral blood smears at one or more time points, 94 (12%) of these harboured gametocytes at presentation while 102 (13%) developed gametocytaemia during treatment.

**Table 2:** Factors associated with gametocytaemia at presentation

	**Gametocytes at presentation**	**No gametocytes at presentation**	**P value of Z score**	**P value (95% CI of odds ratio)**
	**N = 94 (12.8%)**	**N = 639 (81.2%)**		
Duration of illness, days median (IQR)	5 (2-9)	4 (2-6)	0.0303	-
Length of stay in malarious country, days, median (IQR)	29 (19-45)	24 (14-43)	0.3194	-
Maximum parasitaemia, % median (IQR)	0.15 (0.02-1.18)	0.4 (0.05-2.35)	0.0047	-
Anaemia n (%)	57 (61)	235 (36)	-	<0.0005 (1.361-1.998)
Severe anaemia (Hb < 8 g/dl), n (%)	6 (6.4)	16 (2.5)	-	0.045 (1.023-6.352)
Thrombocytopenia, n (%)	52 (55)	495 (76)	-	<0.0005 (0.593-0.860)
Raised bilirubin, n (%)	43 (46)	306 (47)	-	0.702 (0.756-1.208)
Severe malaria, n (%)	35 (37)	318 (49)	-	0.039 (0.570-0.985)
Born in a malarious country, n (%)	59 (63)	390 (60)	-	0.485 (0.743-1.121)
Prophylaxis taken, n (%)	17 (18)	140 (21.4)	-	0.408 (0.524-1.300)

### Gametocytes at presentation

Patients who had gametocytaemia at presentation were more likely to have been ill for longer, to be anaemic and to have a normal platelet count (Table 2). In addition, they were more likely to have a lower parasitaemia and less likely to have severe disease. The presence of gametocytes at presentation did not appear to be influenced by anti-malarial chemoprophylaxis. There was no discernible difference in the rate of gametocytaemia at presentation between those assumed to have partial immunity and those who were malaria-naïve. The time to presentation at a medical facility was no different in naïve patients (Tables 3).

**Table 3:** Factors associated with gametocytaemia at presentation to hospital in malaria naïve patients

	**Gametocytes at presentation**	**No gametocytes at presentation**	**P value of Z score**	**P value (95% CI of odds ratio)**
	**N = 34 (12.6%)**	**N = 235 (87.4%)**		
Duration of illness, days median (IQR)	4 (2-8)	3 (2-6)	0.224	-
Maximum parasitaemia measured (%), median (IQR)	0.3 (0.08 - 3.0)	0.5 (0.02 - 1.75)	0.046	-
Anaemia of any degree, n (%)	24 (71)	99 (40)	-	<0.0005 (1.29 - 2.18)
Severe anaemia (Hb < 8 g/dl), n (%)	2 (6)	7 (3)	-	0.383 (0.428-9.118)
Thrombocytopenia, n (%)	19 (56)	198 (80)	-	0.008 (0.490-0.899)
Raised bilirubin, n (%)	11 (32)	112 (45)	-	0.132 (0.410-1.124)
Severe malaria, n (%)	10 (29)	139 (56)	-	0.010 (0.292-0.846)
Prophylaxis taken, n (%)	9 (27)	51 (21)	-	0.524 (0.662-2.246)

Multivariate logistic regression was performed with five parameters significantly associated with the presence of gametocytes at presentation (in decreasing order of apparent strength of association): anaemia, thrombocytopenia, asexual parasitaemia, duration of illness and severe anaemia (Tables 2 and 3). The continuous variables asexual parasitaemia and duration of illness were converted to binary variables for this analysis using the median as the cut-off in each case (0.4% parasitaemia and 4 days, respectively). Anaemia (OR 2.60, 95% CI 1.59 – 4.24; P <0.001), thrombocytopenia (OR 0.50, 95% CI 0.30 – 0.83; P = 0.007) and duration of illness (OR 1.77, 95% CI 1.10 – 2.82; P = 0.018) remained associated with the presence of gametocytes at the time of presentation.

### Gametocytes developing on treatment

Those who developed gametocytes during treatment were more likely to have presented with a higher parasitaemia and to have at least one marker of severe disease compared to those who did not (Table 4). In addition, these patients tended to have had asexual parasites for longer.

**Table 4:** Factors associated with development of gametocytaemia on treatment in univariate analysis^1^

	**Gametocytes on treatment**	**No gametocytes on treatment**	**P**
	**N = 102 (15.6%)**	**N = 551 (84.4%)**	
Maximum parasitaemia measured (%), median (IQR)	1.0 (0.115-6.325)	0.4 (0.05-2.00)	0.0002*
Duration parasitaemia, days median (IQR)	5 (4-6)	3 (0.05-4)	<0.0001*
Thrombocytopenia proportion	85.3%	75.4%	0.030**
Anaemia proportion	63.7%	31.4%	<0.001**
Severe malaria, n (%)	60 (58.8)	260 (47.2)	0.017**

^1^ 94 individuals who were gametocytaemic at presentation were excluded from this analysis.

* Wilcoxon rank-sum test.

** c^2^ test.

Most patients (643, 83%) were treated with quinine. Artemisinin treatment was postulated to be associated with a lower rate of gametocytaemia development post-treatment. No evidence was found to support this among the 54 patients receiving an artemisinin-containing regimen (p = 0.226). However, this analysis was confounded by the fact that patients treated with artemisinins were more likely to be severe malaria cases (OR 2.80, 95% CI 1.42 - 5.81; p = 0.001) and those with severe malaria were independently more likely to develop gametocytaemia following anti-malarial treatment (Table 4).

Multivariate logistic regression was performed for the parameters showing significant association with development of gametocytaemia during treatment in univariate analysis (Table 4). These were: duration of parasitaemia (median cut-off: 4 days), anaemia, thrombocytopenia, severe malaria and asexual parasitaemia. Of these parameters, duration of parasitaemia (OR 1.48, 95% CI 1.25 – 1.75; P < 0.001) and the presence of any degree of anaemia (OR 3.13, 95% CI 1.91 – 5.13; P < 0.001) remained significantly associated with development of peripheral gametocytaemia.

## Discussion

Patients with gametocytes seen on their peripheral blood film fall into two groups: those who have gametocytaemia at presentation and those who develop gametocytaemia during treatment. It was postulated that those who have gametocytaemia at presentation would be more likely to have been ill for longer; analysis of the data supported this, in both the duration of symptoms and the haematological findings. These patients were more likely to be anaemic (associated with longer illness) and less likely to have thrombocytopenia (associated with acute illness) than those who did not have gametocytes at presentation. The data also suggest that despite the chronicity of their illness, these patients were less unwell; they tended to have a lower parasitaemia and fewer of them had any markers of severe malaria.

There are two principal explanations for the ability of some patients to tolerate malarial infection for longer without becoming unwell. One is the use of partially effective infrequent anti-malarial chemoprophylaxis, which may attenuate an infection without clearing it. The other is previous exposure to malaria, which may induce acquired immunity. There was no demonstrable association between prophylaxis use (either complete or incomplete) and gametocytaemia at presentation. It is well documented that immunity to *P. falciparum* develops among children who live in highly endemic areas and survive repeated infections [11,12]. It may be reasonable to assume that patients who grew up in malaria-endemic parts of the world and then migrated to the UK may have some residual, but incomplete, immunity to the disease and may therefore be more likely to tolerate a subsequent infection. However, there was no discernible relationship between being born in an endemic country and having gametocytes at presentation.

Patients who have not grown up in an endemic area are usually considered to be malaria-naïve. Intuitively, this group may be considered at greater risk of severe disease and less likely to tolerate malaria infection. However, this group had a similar rate of gametocytaemia at presentation and were no more likely to have markers of severe disease. In this group, factors associated with gametocytaemia (severity of disease, anaemia, thrombocytopenia, etc) mirrored the findings from studies in endemic areas. In a three-centre study, gametocytaemia at presentation was seen in 35% of cases from Thailand, 37% of cases from The Gambia and 26% of cases from Tanzania [13]. The rate of gametocytaemia reported here for the naive group was lower than all of these estimates at 12.6%. Time to presentation was no different in those who did and did not have gametocytaemia at presentation in this group, suggesting that gametocytaemia at presentation is not related to a delay in presentation to medical services.

The data indicate that although previous exposure may be important in determining which patients present with gametocytaemia, this is not the full explanation. It seems conceivable that some form of innate immunity may also play a role in modulating the nature of the illness and the likelihood of gametocytes being present early in the clinical disease. A number of factors have been associated with the potential to protect against severe complications of malaria, but contribute towards anaemia, such as sickle cell trait [14]. However, what has been observed here may be independent of such factors. These findings are consistent with the limited published data on immunity to circulating sexual stages of *P. falciparum*, which indicate that previous infection history is a much less critical factor than it is for immune modulation of asexual parasitaemia [9,12,15].

The emergence of circulating gametocytes in patients receiving anti-malarial treatment has been associated with the time taken to clear asexual parasites from the peripheral circulation [16]. The data presented here corroborate this, in that patients who had a longer period of parasitaemia, who started with a higher parasitaemia and who had severe malaria were more likely to develop gametocytes. High parasitaemia and severe malaria are not independent variables and, unsurprisingly, in multivariate analysis severe malaria did not remain significantly associated with gametocyte emergence during treatment. Artemisinin compounds clear parasitaemia more rapidly than other anti-malarial drugs and are also gametocytocidal [17,18]. It was anticipated that treatment with artemisinins would reduce the prevalence of gametocytaemia. This was not supported by the data, although the analysis was confounded by the fact that artemisinin therapy was more likely to be used for patients with higher parasite counts.

## Conclusion

No evidence was found that gametocyte carriage among adult malaria patients in the UK was related to previous exposure to malaria. This suggests that acquired immunity alone is not the sole determinant of gametocyte emergence in clinical cases.

## Competing interests

The authors declare there are no financial relationships with any organisations that might have an interest in the submitted work, nor other relationships or activities that could appear to have influenced the submitted work or its interpretation.

## Authors’ contributions

Data collection was performed by CHR, MA, EZ, SB, SW and PLC. Analysis was performed by all authors. The manuscript was drafted by CHR, and revised by MA, CJS, and TD. All authors read and approved the final manuscript.
